# Culicoids as Vectors of Schmallenberg Virus

**DOI:** 10.3201/eid1807.120385

**Published:** 2012-07

**Authors:** Lasse Dam Rasmussen, Birgit Kristensen, Carsten Kirkeby, Thomas Bruun Rasmussen, Graham J. Belsham, René Bødker, Anette Bøtner

**Affiliations:** Technical University of Denmark, Kalvehave, Denmark (L.D. Rasmussen, T.B. Rasmussen, G.J. Belsham, A. Bøtner);; Technical University of Denmark, Copenhagen, Denmark (B. Kristensen, C. Kirkeby, R. Bødker)

**Keywords:** Orthobunyavirus, Schmallenberg virus, Culicoides spp., RT-qPCR, biting midge vector, viruses, Denmark, Germany, arthropods, culicoids

**To the Editor:** In autumn 2011, an unidentified disease of livestock was reported on both sides of the Dutch–Germany border. By using metagenomics, the etiologic agent of this disease was identified as a novel orthobunyavirus and named Schmallenberg virus (SBV) (*1*). Other members of the genus *Orthobunyavirus* (e.g., Akabane virus) are widespread in Africa and Asia; biting midges (*Culicoides* spp.) and mosquitoes are responsible for transmitting these viruses. Hence, we reasonably assumed that European culicoids might be responsible for transmitting SBV within Europe. We present evidence that culicoids captured October 2011 in Denmark contained SBV RNA and most likely are vectors for this agent.

In autumn 2011, culicoids were collected from several sites within Denmark. One site, a chicken farm in Hokkerup (Figure A1), was selected for study because of its location close (6 km) to the German border and proximity (<10 km) to an SBV-infected sheep farm in Germany, as reported on March 9, 2012, by the Friedrich Loeffler Institute surveillance website (www.fli.bund.de). The culicoids were collected during October 14–16 by using a Mosquito Magnet Independence trap (Mosquito Magnet, Lititz, PA, USA) baited with carbon dioxide and octenol. Midges were sorted manually into 91 specimens of the *C. obsoletus* group (comprising *C. obsoletus*, *C. chiopterus*, *C. dewulfi*, and *C. scoticus*) and 17 of the *C. punctatus* sensu stricto group, then stored at −20°C.

Pools of culicoids were homogenized in water (100 µL) by using a 3-mm stainless steel bead (Dejay Distribution Ltd., Launceston, UK) in a TissueLyser II (QIAGEN, Hilden, Germany) for 1 min at 25 Hz (*2*). After homogenization, additional water (100 µL) was added to the samples, and then the mixture was centrifuged at 3,000 × g for 5 min. Nucleic acids were extracted from the supernatant (100 µL) by using a MagNA pure LC Total Nucleic Acid Isolation Kit on a MagNA pure LC (Roche Diagnostics, Basel, Switzerland) and eluted in water (50 µL).

Two separate 1-step reverse transcription quantitative PCRs (RT-qPCRs), targeting the L segment and the S segment of SBV RNA, were performed according to protocols provided by the Friedrich Loeffler Institute in Germany (*1*) on the extracted nucleic acids by using a Mx3005p qPCR system (Agilent Technologies, Palo Alto, CA, USA). Another RT-qPCR targeting ruminant β-actin mRNA was performed as an internal endogenous control (*3*).

Two of 22 pools tested strongly positive for the large (L) and small (S) segments of SBV RNA. Each positive sample was derived from 5 midges of the *C. obsoletus* group. One pool produced cycle threshold (C_t_) values of 26.4 and 24.5 (in the L segment– and S segment–specific assays, respectively), whereas the second positive pool gave C_t_ values of 28.8 (L segment) and 27.6 (S segment). These pools were negative for the internal endogenous control that targeted the bovine/ovine β-actin mRNA. This result makes it unlikely that the detection of SBV RNA within the midges resulted from recent blood meals from infected animals remaining within the culicoids and suggests the virus has replicated within the midges. The PCR amplicons (145 bp; Figure) from the L segment–specific RT-qPCR were sequenced by using BigDye 1.1 chemistry on an ABI 3500 Genetic Analyzer (Applied Biosystems, Foster City, CA, USA). The sequences of 80 bp from the amplicons, excluding the primer sequences, had 100% identity with the expected region of the SBV segment L (*1*).

**Figure:**
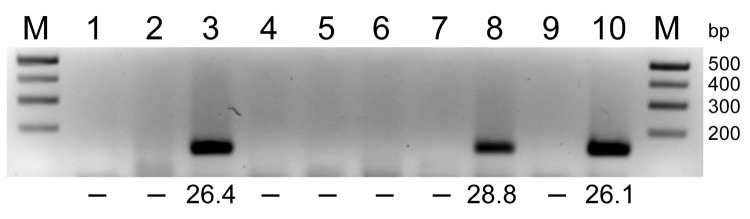
RNA extracted from pools of *Culicoides obsoletus* group midges was tested in 1-step reverse transcription quantitative PCRs (RT-qPCRs) for the Schmallenberg virus large segment, and the products were analyzed by agarose gel electrophoresis. Lanes 1–8, *C. obsoletus* group midge pools 1–8; lanes 9–10; negative and positive controls, respectively. Numbers below lanes are cycle threshold values from RT-qPCRs; –, no value. M, size marker. Amplicons (145 bp) from positive pools were extracted and sequenced.

Reported C_t_ values generated by using the same assays from blood of naturally infected cattle were 24–35 (*1*). Usually, ≈100 µL of bovine/ovine blood is used for virus detection, whereas <1 µL of blood remains in a midge after a blood meal. This uptake of blood should therefore lead to a C_t_ value that is at least 6–7 units higher (≈100-fold lower level of RNA) when a single midge is tested by RT-qPCR (*4*). Thus, even if all 5 culicoids in a pool had recently taken a blood meal from a viremic animal, the C_t_ values observed here strongly suggest replication of SBV within the *C. obsoletus* group midges. However, in principle, other hosts of SBV could have a much higher level of viremia than cattle and could provide the levels of SBV RNA detected. *C. punctatus* s.s. midges cannot be ruled out as a possible vector of SBV because of the limited number of insects tested.

Our study demonstrates the presence of SBV RNA in *C. obsoletus* group midges caught in Denmark during October 2011. The low C_t_ values (i.e., high SBV RNA levels) and the absence of ruminant β-actin mRNA in these samples strongly suggest that SBV replicates in these midges and hence that the *C. obsoletus* group midges are natural vectors for this virus.

## Figures and Tables

**Figure A1:**
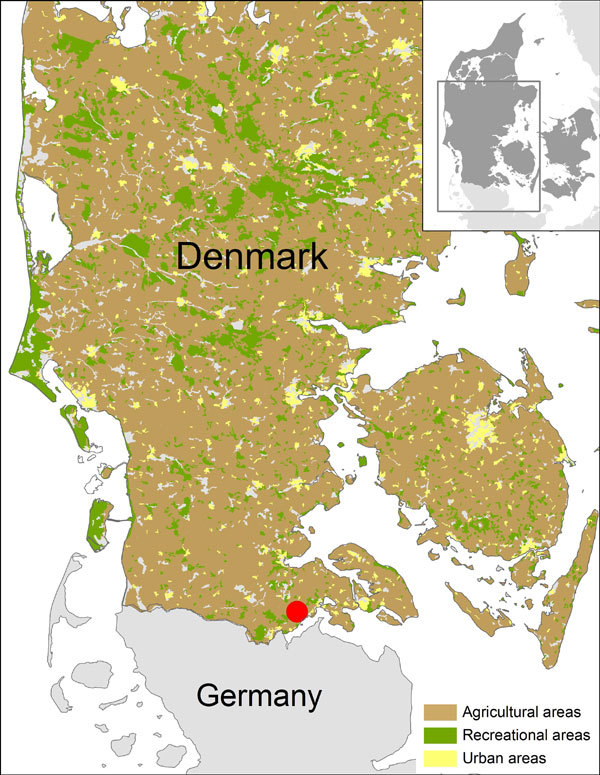
Location of trap site with culicoids positive for Schmallenberg virus (red dot), Hokkerup, Denmark, 2011.
